# The in vitro effects of interferon-gamma, alone or in combination with amphotericin B, tested against the pathogenic fungi *Candida albicans* and *Aspergillus fumigatus*

**DOI:** 10.1186/s13104-017-2696-4

**Published:** 2017-08-01

**Authors:** Moufid El-Khoury, Rogine Ligot, Simon Mahoney, Colin M. Stack, Gabriel G. Perrone, C. Oliver Morton

**Affiliations:** 1 School of Science and Health, Western Sydney University, Building 21 Campbelltown Campus, Narellan Road, Campbelltown, NSW 2560 Australia; 2 School of Science and Health, Western Sydney University, Locked Bag 1797, Penrith, NSW 2571 Australia

**Keywords:** Amphotericin B, Interferon-gamma, Antifungals, Aspergillosis

## Abstract

**Objective:**

Recent studies into the antifungal activity of NK-cells against the *Aspergillus fumigatus* have presented differing accounts on their mode of antifungal activity. One of these mechanisms proposed that NK-cells may kill the fungus via the direct effects of exposure to Interferon gamma (IFN-γ).

**Results:**

In this study we investigated the direct antifungal effects of recombinant human IFN-γ against a range of pathogenic fungi by measuring cellular damage using an XTT-based assay and cell viability through plate counts. It was found that 32 pg/ml of IFN-γ exhibited a significant but small antifungal effect on *A. fumigatus* (p = 0.02), *Aspergillus flavus* (p = 0.04) and *Saccharomyces cerevisiae* (p = 0.03), inhibiting growth by 6, 11 and 17% respectively. No significant inhibitory effects were observed in *Candida* species (p > 0.05 for all species tested) or *Cryptococus neoformans* (p = 0.98). Short term exposure (3 h) to a combination of amphotericin B (1 µg/ml) and IFN-γ (32 pg/ml) increased the effectiveness of amphotericin B against *A. fumigatus* and *S. cerevisiae* but not *Candida albicans*. These data suggest that IFN-γ does not possess strong antifungal activity but can enhance the effect of amphotericin B under some testing conditions against *Aspergillus* species.

## Introduction

Invasive aspergillosis (IA) is a life-threatening fungal disease of individuals undergoing immunosuppressive treatments such transplants or those with predisposing conditions such as advanced AIDS [1, 2]. Despite current treatment methods the mortality rate is as high as 40% and the number of effective antifungal agents available is limited due to the similarity between fungal and mammalian cells. Antifungal drug resistance, particularly to azoles, is also increasing which has made it imperative to identify novel antifungal therapies and drug targets [3].

The immune response to IA has received a lot of attention and it has been shown that immune reconstitution and immunomodulation are necessary for effective treatment of IA [4]. In neutropenic patients the activity of cytokines is essential for determining the outcome of IA, with proinflammatory cytokines being of prime importance. Interferon-gamma (IFN-γ) is a proinflammatory cytokine with an important role in immunity against IA, and in neutropenic mice its secretion by natural killer cells (NK-cells) was found to be essential for defense against the fungus [5].

The importance of IFN-γ has led to its use as a therapeutic intervention in IA, with several studies assessing the potential of IFN-γ alone or in combination with antifungal drugs to treat IA [6, 7]. In a review of case studies it was found that immunotherapy with IFN-γ could restore immune function and aid in the treatment of fungal sepsis [8]. In experimental models of cryptococcosis the administration of IFN-γ was weakly effective but it was highly effective in combination with amphotericin B [9]. Thus combined treatment with antifungals and immunotherapy may be a promising strategy to improve treatments against IA. Interestingly, data from a study of the in vitro interaction between NK-cells with *Aspergillus fumigatus* indicated that IFN-γ might be responsible for NK-cell mediated killing of *A. fumigatus* [10]. However, this antifungal activity of IFN-γ was not observed in another interaction study between NK-cells and *A. fumigatus* [11], which implicated perforin and granzyme in the killing of *A. fumigatus* by NK-cells.

## Main text

The purpose of this study was to answer the question arising from these interactions studies; does IFN-γ have significant antifungal activity? Furthermore, this study also examined if addition of IFN-γ could enhance the activity of antifungal drugs in vitro?

### Organisms and strains

The strains used in this study were *Candida albicans*, *Candida glabrata, Candida krusei, Candida parapsilosis, Cryptococcus neoformans, A. fumigatus, Aspergillus flavus* and *Aspergillus clavatus;* clinical isolates obtained from the culture collection at Westmead Hospital (Sydney, Australia). *Saccharomyces cerevisiae* strain BY4742 was used in this study (Thermo Fisher). The fungi were maintained on potato dextrose agar and defined inocula were prepared as previously described [12].

### Measurement of fungal metabolic activity

The XTT (2,3-Bis-(2-Methoxy-4-Nitro-5-Sulfophenyl)-2H-Tetrazolium-5-Carboxanilide) metabolic assay was used to assess metabolic inhibition caused by recombinant IFN-γ (R & D Systems) as previously described [10]. Assays were conducted in RPMI 1640 media with glucose. Samples (5 × 10^5^ CFU/ml) were incubated at 30 °C for *S. cerevisiae* or 37 °C for the other fungi for 3 h with 0, 4, or 32 pg/ml of IFN-γ followed by addition of reagents to give final concentrations of 0.3 mg/ml XTT and 75 µM menadione in each sample. The doses were based on a baseline IFN-γ level in serum (4 pg/ml) and a minimum inhibitory level against *A. fumigatus* (32 pg/ml) [10]. The samples were incubated for a further 1.5 h. The supernatant from each sample, following centrifugation, was measured at both 450 and 492 nm using a spectrophotometer [10].

The effects of treatment with a combination of IFN-γ and amphotericin B were measured by different methods.

### CFU counts

Cultures containing final concentrations of approx. 5 × 10^4^ colony forming units (C.F.U.)/ml of *S. cerevisiae*, *C. albicans* and *A. fumigatus* with combinations of IFN-γ (32 pg/ml) and amphotericin B (1 or 2 µg/ml) were prepared. These fungi were tested because *A. fumigatus* and *S. cerevisiae* were sensitive and *C. albicans* was relatively resistant to IFN-γ in XTT assays. These were incubated for 3 h to avoid hyphal development in *A. fumigatus*, after incubation serial dilutions of each culture were prepared and plated onto YPD agar; these were incubated at 30 °C for *S. cerevisiae* and 37 °C for the other fungi for two days followed by counting of C.F.U.

### MIC determinations

Yeast Sensititre Plate YO10 (Thermo Scientific) is a microdilution method that was used in this study to determine the MIC of common antifungal drugs against selected fungi. The tests were performed as per the manufacturer’s instructions for plates without IFN-γ. For plates including IFN-γ; IFN-γ was added to the YeastOne inoculum broth to yield a final concentration of 32 pg/ml. Fungal cells were then added to the broth as described in the manufacturer’s instructions to achieve an organism density of 1.5–8 × 10^3^ cells/ml; 100 µl of broth containing cells and IFN-γ was added to each well of the YO10 Sensititre plate. After inoculation the plates were incubated at 37 °C for 24 h. These tests were performed three times. The YO10 Sensititre plate contains the following drugs (concentration range): Amphotericin B (0.12–8 µg/ml), Anidulafungin (0.015–8 µg/ml), Caspofungin (0.008–8 µg/ml), Fluconazole (0.12–256 µg/ml), 5-Flucytosine (0.06–64 µg/ml), Itraconazole (0.015–16 µg/ml), Micafungin (0.008–8 µg/ml), Posazonazole (0.008–8 µg/ml), Voriconazole (0.008–8 µg/ml).

### Statistical analysis

**Fig. 1:**
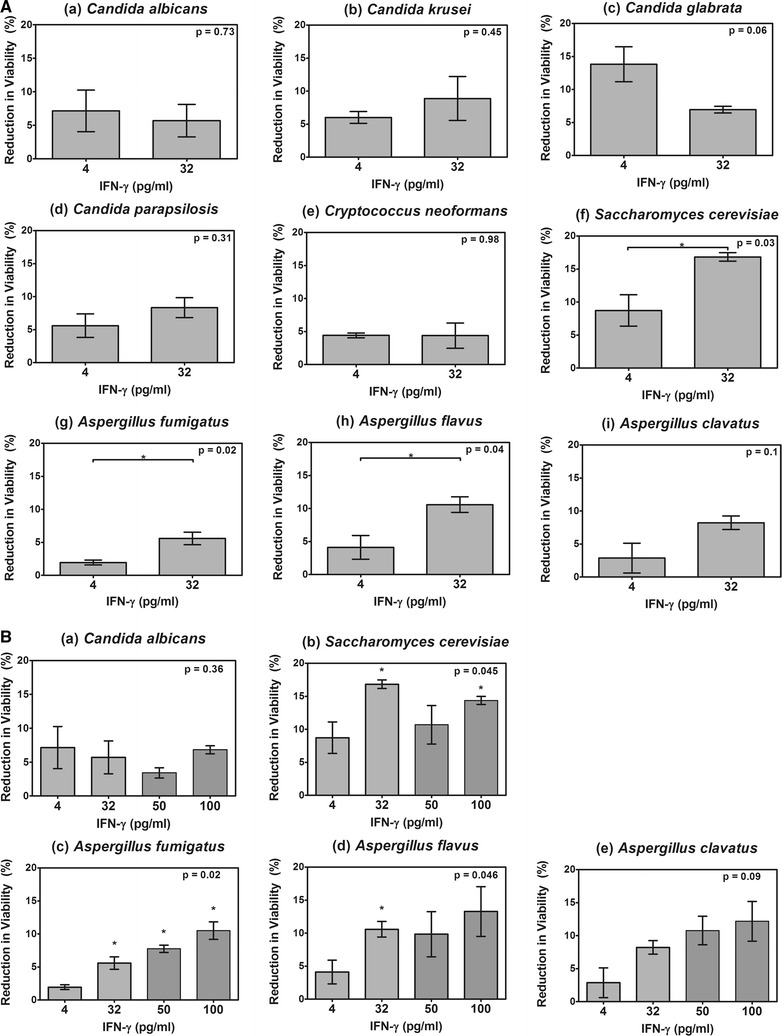
**A** Measurement of inhibition caused by treatment with IFN-γ on several pathogenic fungi using the XTT assay. The results were expressed as the percentage of the metabolic activity of treated cells compared to untreated cells. The absorbance reading at OD_492_ for the untreated control for each species was taken as 100%; the mean OD_492_ value was 0.44 (±0.07). The data shown are means and standard errors of the growth inhibition (treated/untreated control) from three replicate experiments. Data were analysed by t-test to compare low to high doses of IFN-γ (*p < 0.05, **p < 0.01, ***p < 0.001), p-values for each test are shown in the figure. **B** Measurement of inhibition caused by incubation of several pathogenic fungi with IFN-γ using the XTT assay over an extended dose range. No significant differences in fungal survival were found when treatment with 32 pg/ml to treatment with 50 or 100 pg/ml of IFN-γ were compared. Data in 1B were analysed by one-way ANOVA (p-value shown in the figure) and Dunn’s post-test (p-values shown as asterisks) to compare the range of doses of IFN-γ (*p < 0.05, **p < 0.01, ***p < 0.001). *Asterisks* indicate that doses are significantly different to the lowest dose

**Fig. 2:**
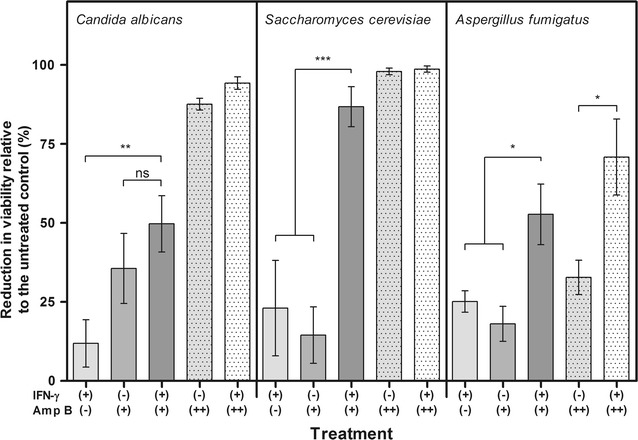
The effect of short term exposure to IFN-γ alone or in combination with amphotericin B on selected fungi. The addition of (I+; 32 pg/ml) and amphotericin B (A+; 1.0 µg/ml. A++; 2.0 µg/ml) were tested on *C. albicans*, *S. cerevisiae* and *A. fumigatus*. The effect was measured as CFU following a three hour incubation relative to the untreated control samples (100%) were used to calculate the growth inhibition **a** *S. cerevisiae* 3.1 × 10^4^/ml (p < 0.0001); **b** *C. albicans* 3.5 × 10^4^/ml (p = 0.0011) and **c** *A. fumigatus* 4.1 × 10^4^/ml (p = 0.002). The data presented are means and standard errors of growth inhibition (treated/untreated) from, three replicate experiments. The data was analysed by one-way ANOVA and Dunn’s multiple comparison test to compare all treatments (*p < 0.05, **p < 0.01, ***p < 0.001). Three independent experiments were conducted for each treatment

The effects IFN-γ on pathogenic fungi were analysed by two-tailed *t* test (XTT assay, Fig. 1A) or one–way ANOVA with Dunn’s post-test (Figs. 1B, 2) using Graphpad Prism Version 5.02 for Windows (Graphpad Software, San Diego, CA, USA).

This study examined the antifungal potential of IFN-γ alone or in combination with amphotericin B against pathogenic fungi in vitro. The data for short term exposure (3 h) (Figs. 1A, 2) was compatible with in vivo studies showing that the combination could be more effective than a single agent [9].

### The direct antifungal effects of IFN-γ on pathogenic fungi

Testing the antifungal activity of IFN-γ on nine fungi revealed significant sensitivity in *A. fumigatus* (p = 0.02), *A. flavus* (p = 0.04) and the non-pathogen *S. cerevisiae* (p = 0.03) to 32 pg/ml (Fig. 1A), which agreed with previous findings for *A. fumigatus* [10] and *C. albicans* [13]. This quantity is higher than the plasma IFN-γ concentrations of ca. 20 pg/ml reported for individuals with severe infection [14], however during immunotherapy serum levels of IFN-γ can rise to over 100 pg/ml for periods of up to 12 h after administration of a dose of 100 μg/m^2^ [15], which is the dosage used in cases using IFN-γ to treat IA [8]. The other fungi that were tested were unaffected by these concentrations of IFN-γ and were even resistant to higher concentrations (up to 100 pg/ml) (Fig. 1B). No significant differences were observed between treatment with 32 pg/ml and treatments with 50 pg/ml or 100 pg/ml; although there was a trend for increased killing with increased dose for *A. fumigatus* and *A. clavatus* (Fig. 1B).

The limited antifungal toxicity of IFN-γ suggests that it is not the sole mechanism of NK-cell mediated antifungal activity and that perforin-mediated cytotoxicity is a more convincing candidate [11]. The perforin–granzyme system is an established mechanism of inducing death in target cells, whereas IFN-γ is an established immunoregulator. There is evidence that NK cell-derived IFN-γ is an essential component of host defence against invasive aspergillosis in neutropenic mice [5]. This was determined when depletion of NK-cells from murine lungs resulted in increased fungal load independent of the presence of T and B cells [5]. The role of IFN-γ in these mice was proposed to be due to stimulation of macrophages and cytokine production that modulated the immune system to enable an effective antifungal response, which was also a component of in NK-cell mediated activity against *Candida* [13]. However, it has been observed that IFN-γ expression by NK-cells was reduced by the presence of *A. fumigatus* [11] and *Rhizopus oryzae* [16], indicating that the role of IFN-γ in NK-cell interactions with moulds may be complex.

### The effects of combined amphotericin B and IFN-γ treatment

A combination of amphotericin B and IFN-γ induced greater direct antifungal activity than amphotericin B or IFN-γ alone (Fig. 2); this was particularly significant for *S. cerevisiae* (p < 0.001) and *A. fumigatus* (p = 0.002). *A. fumigatus* showed less sensitivity than the other two fungi after 3 h exposure to amphotericin B (2 µg/ml); but showed a significant effect when exposed to combinations of IFN-γ (32 pg/ml) and amphotericin B (1 µg/ml and 2 µg/ml).

Amphotericin B was chosen since it is an important fungicide for treating invasive mycoses and because it is a membrane targeting molecule. In the absence of immune cells it was interesting that IFN-γ appeared to enhance the activity of amphotericin B against the fungi tested (Fig. 2). Amphotericin B is proposed to act by causing the formation of pores in target membranes. A molecule with a similar mode of action is the toxin listeriolysin O (LLO) from *Listeria monocytogenes*; the activity of LLO against human cells has been found to be enhanced by IFN-β [17]. This could provide an avenue to further understand the increased activity of IFN-γ and amphotericin B against some fungi.

However, these data were not supported by combining IFN-γ with the antifungals on sensititre YO10 plates (Table 1). There was no significant change in the MICs for *A. fumigatus* or *C. albicans* other than MIC for fluconazole against *C. albicans* was reduced from 16 to 4 µg/ml. This difference between C.F.U. counts and Sensititre data is probably because the Sensititre readings were taken after 24 h growth, in that period any influence of IFN-γ may have been masked by the drug. This is similar to the effect seen for *S. cerevisiae* where treatment with 2 µg/ml amphotericin B caused complete inhibition as did treatment with 2 µg/ml amphotericin and IFN-γ; therefore no differences could be detected between the two treatments (Fig. 2).

**Table 1:** MIC data for *Aspergillus fumigatus* and *Candida albicans* inoculated into Sensititre YO10 plates plus or minus 32 pg/ml interferon-γ

Drug	*A. fumigatus*		*C. albicans*	
	Plus IFN-γ	Minus IFN-γ	Plus IFN-γ	Minus IFN-γ
	MIC (µg/ml)	MIC (µg/ml)	MIC (µg/ml)	MIC (µg/ml)
Anidulafungin	2	2	0.015	0.03
Micafungin	1	1	0.015	0.015
Caspofungin	2	2	0.06	0.12
Flucytosine	64	64	<0.06	<0.06
Posaconazole	0.5	1	1	1
Voriconazole	0.25	0.5	0.12	0.25
Itraconazole	0.25	0.25	0.25	0.5
Fluconazole	256	256	*4*	*16* ^a^
Amphotericin B	0.5	1	0.5	0.5

^a^MIC values in italic indicate a significant difference

In answer to the initial questions posed in this study: IFN-γ is weakly fungicidal against some *Aspergillius* species and *S. cerevisiae*. This antifungal activity is not sufficient to suggest that it would be the primary factor in NK-cell mediated fungal killing but may play a role in a more complex process that would require further study including NK-cells.

Secondly there is evidence that IFN-γ can enhance the activity of amphotericin B in vitro but this varied between fungi and experimental conditions. The data from this study do not contradict the paradigm that IFN-γ in antifungal therapy acts as an immunomodulator [8]. However, the differential responses of the fungi tested against combination therapy with IFN-γ and amphotericin B indicates a path to study differences in cell wall and membrane composition that might be exploited for the development of targeted antifungals.

## Limitations

To conclusively determine the mechanism of NK-cell activity against fungi is beyond the scope of this study. We cannot determine the complete significance of IFN-γ in the context of complex intercellular interactions but show that it is not the sole mechanism of antifungal activity.

Extended exposure times and high drug concentrations in the Sensititre tests may have masked the effect of combined treatment with IFN-γ and antifungals. Much more detailed study is required to fully examine the nature of any enhancement of amphotericin B activity by IFN-γ.

## Abbreviations

MIC: minimum inhibitory concentration
IFN-γ: interferon gamma
LLO: listeriolysin O
